# Venomous Animal Bites and Stings in Active Component U.S. Service Members, 2008–2023

**Published:** 2025-08-20

**Authors:** Ralph A. Stidham, José M. Jimenez, Sithembile L. Mabila

**Affiliations:** Epidemiology and Disease Surveillance, U.S. Army Public Health Command, West: Dr. Stidham; Department of Health and Business Administration, Army–Baylor Graduate Program, U.S. Army Medical School of Excellence, Joint Base San Antonio–Fort Sam Houston: MAJ Jimenez; Epidemiology and Analysis Branch, Armed Forces Health Surveillance Division, Public Health Directorate, Defense Health Agency, Silver Spring, MD: Dr. Mabila

## Abstract

This study characterizes all medically diagnosed bites and stings in active component service members (ACSMs) from snakes, venomous fish, other venomous marine animals, arthropods, and insects identified through an evaluation of medical data from the Defense Medical Surveillance System (DMSS). Incident trends were determined from 2008 through 2023, and incidence rates (IRs) and incidence rate ratios (IRRs) were calculated. In total, there were 42,552 venomous bite and sting medical encounters among 39,201 ACSMs, resulting in an IR of 19.3 cases per 10,000 person-years (p-yrs) during the surveillance period. Most cases occurred in men ages 20-34 years, non-Hispanic White individuals, Army service members, and junior enlisted ranks. IRs were elevated in female (25.0 per 10,000 p-yrs), youngest (<20 years, 24.5 per 10,000 p-yrs), and Coast Guard or U.S. Public Health Service (23.4 per 10,000 p-yrs) ACSMs. Arthropods were the primary source (75.0%) of stings and bites. IRR calculations suggest that women had a 37.0% higher risk than men. These study findings demonstrate the continuing susceptibility of ACSMs to venomous encounters and the importance of targeted prevention strategies, training, and comprehensive medical support to maintain force readiness.

Snake, marine animal (e.g., jellyfish, sea anemones), arthropod (e.g., scorpions, spiders), and insect (e.g., bees, wasps) envenomation can cause a range of adverse events—from mild irritation and limited necrosis of tissue to systemic reaction, renal, hepatic, cardiac or respiratory failure, failure, and death—with serious implications for the health and operational readiness of military personnel. ^1^

Approximately 5.4 million people worldwide are bitten by snakes each year, with 1.8 to 2.7 million cases of envenoming, with most in Africa, Asia, and Latin America. Between 81,410 and 137,880 people die each year as a result of snake bite. ^2^ Marine envenomation is generally not medically significant and includes mild stings, bites, abrasions, and lacerations, with jellyfish stings the most frequent type worldwide, accounting for more than 150 million stings annually ^4^ ; notable exceptions include lethal box jellyfish ( *Chironex fleckeri* ) or sea wasp stings, Irukandji jellyfish ( *Carukia barnesi* ) and blue bottle ( *Physalia* ) stings, and sea snake bites ( *Hydrophiinae* ) that occur most frequently in Australian and Pacific waters. ^5^ Arthropod envenomation accounts for a higher percentage human morbidity and mortality than snake or marine envenomation. ^3^ Medically significant scorpion sting encounters involve more than 1 million people annually, but with a low fatality rate. ^6^ Species that present common stinging threats include honeybees ( *Apidae* ), wasps, yellowjackets, hornets ( *Vespidae* ), and ants ( *Formicidae* ). ^7^ Allergic reactions to these hymenopteran insect stings are common, but in some cases, they can produce systemic allergic reactions that may lead to fatal anaphylaxis.

**What are the new findings?**

Venomous bites and stings are a persistent health concern for active component service members. Arthropods are the most common culprit, but risks vary by sex, age, and military occupation. This report also reveals that younger service members and women are disproportionately affected.

**What is the impact on readiness and force health protection?**

Venomous bites and stings, while often non-fatal, represent a persistent health risk that affects U.S. military personnel readiness worldwide. Higher incidence among specific demographics (e.g., women and younger personnel) and occupations (e.g., veterinary) necessitates targeted force health protection measures. Further efforts to improve service member awareness, as well as preventive measures, may help reduce sequelae and mitigate impacts on military readiness.

Previous studies specific to active component service members (ACSMs) have focused mainly on arthropod and snake envenomations. From 1990 through 1997, 728 ACSMs were hospitalized for arthropod or snake envenomations, with average hospitalization of 4.2 days. ^8^ A recent 5-year surveillance study of snakebite envenomation (SBE) among U.S. active and reserve component service members ^9^ revealed a total of 345 SBE diagnoses from 2016 through 2020. Most SBEs were among ACSMs, who were relatively young (ages 20-29 years) and in combat-specific or repair and engineering occupations.

The objective of this study was to characterize all medically diagnosed bites and stings in ACSMs from snakes, arthropods, venomous fish, and other venomous marine animals that were identified through an evaluation of medical data from the Defense Medical Surveillance System (DMSS). This review also provides a summary of bites and stings service members sustained, by demographic and military characteristics including combatant command and location where bites and stings occurred and were treated.

## Methods

This retrospective cohort study included all ACSMs of any branch of the U.S. Armed Forces during the surveillance period, from January 1, 2008 through December 31, 2023. Data were queried from the DMSS. Demographic information on age, sex, service, race and ethnicity, rank, and military occupation were included. International Classification of Diseases, 9th and 10th Revisions (ICD-9/ICD-10) diagnostic codes were used to define bites and stings from venomous animals (ICD-9: 989.5, E905.0; ICD-10: T63.0–T63.6, T63.8, T63.9) **(Table 1)** . From October 2015 through 2023, ICD-10 diagnostic codes, which are more exact than ICD-9 codes, were used to delineate bite and sting incident cases from specific categories of venomous animals.

**TABLE 1.** Diagnostic Codes to Identify Venomous Bites and Stings

ICD-10	ICD-9	Description
—	989.5	Toxic effect of venom
—	E905.0	Venomous snakes and lizards causing poisoning and toxic reactions
T63.0	—	Toxic effect of snake venom
T63.1	—	Toxic effect of venom of other reptiles
T63.2	—	Toxic effect of venom of scorpion
T63.3	—	Toxic effect of venom of spider
T63.4	—	Toxic effect of venom of other arthropods
T63.5	—	Toxic effect of contact with venomous fish
T63.6	—	Toxic effect of contact with other venomous marine animals
T63.8	—	Toxic effect of contact with other venomous animals
T63.9	—	Toxic effect of contact with unspecified venomous animal

Abbreviation: ICD-10, International Classification of Diseases, 10th Revision; ICD-9, International Classification of Diseases, 9th Revision.

ACSMs with either 1 inpatient or out-patient medical encounter for a venomous animal bite case-defining code in any diagnostic position were considered a case. The date of the first case medical encounter at military hospitals or clinics as well as private sector facilities was used as the incident date. Cases were counted once per year.

Person-time contributions for each service member were determined from January 1, 2008 through December 31, 2023. Person-time for each ACSM was calculated until the individual left active component service or were lost to follow-up, or the surveillance period ended, whichever occurred first. Since cases were counted once per year, person-time was not changed at the time of incident encounter.

Incident cases of venomous bites were determined according to key demographic variables. Incident cases from October 2015 through 2023 were further stratified by type of venomous animal, as ICD-10 codes provide more specificity than cases that were identified from ICD-9 codes in prior years. Crude incidence rates (IRs) were calculated as incident venomous bites per 10,000 person-years (p-yrs). The Poisson regression was used to calculate incidence rate ratios (IRRs) and 95.0% confidence intervals (CIs). The least at-risk sub-group of each demographic variable was selected as the reference group. All analyses were conducted using SAS Enterprise Guide (version 8.3).

## Results

During the 16-year surveillance period, a total of 42,552 incident cases of venomous bites and stings were diagnosed among 39,201 ACSMs. Case-defining medical encounters with ICD-9 diagnostic codes identified 19,626 incident cases from 2008 through 2023, and ICD-10 diagnostic codes identified 22,926 incident cases from October 2015 through 2023.

Approximately 80.0% (n=33,887) of cases were among male service members. Nearly 70.0% (n=29,370) of cases occurred in those aged 20-34 years, 63.0% (n=26,879) occurred in non-Hispanic White individuals, 44.0% (n=18,930) were among junior enlisted ranks (E1–E4), and 40.0% (n=17,104) occurred in Army members **(Table 2)** . ACSMs in the repair / engineering and communications / intelligence occupational groups were most often affected by bites and stings, comprising nearly half of all bite and sting occurrences during the surveillance period **(Table 2)** .

**TABLE 2.** Demographics and Military Characteristics of Venomous Bite and Sting Cases, Active Component, U.S. Armed Forces ^a^ , 2008–2023

Characteristic	No.	IR (per 10,000 person-years)	IRR	95% CI Lower Limit	95% CI Upper Limit
Total	42,552	19.3			
Sex					
Male	33,887	18.2	Reference	—	—
Female	8,665	25.0	1.4	1.3	1.4
Age, y					
<20	3,585	24.5	1.3	1.3	1.4
20–24	13,280	19.0	1.0	1.0	1.1
25–29	9,604	18.4	Reference	—	—
30–34	6,486	18.6	1.0	1.0	1.0
35–39	4,973	19.1	1.0	1.0	1.1
40–44	2,859	19.6	1.0	1.0	1.1
45+	1,765	21.3	1.2	1.1	1.2
Race and ethnicity					
White, non-Hispanic	26,879	20.9	1.4	1.4	1.5
Black, non-Hispanic	5,030	14.5	Reference	—	—
Hispanic	5,657	17.5	1.2	1.2	1.3
Other	4,986	19.8	1.4	1.3	1.4
Service branch					
Army	17,104	21.3	1.6	1.5	1.6
Navy	7,055	13.5	Reference	—	—
Air Force	9,943	19.2	1.4	1.4	1.5
Marine Corps	6,987	23.2	1.7	1.7	1.8
Coast Guard, USPHS	1,463	23.4	1.7	1.6	1.8
Rank					
E1–E4	18,930	20.0	1.1	1.1	1.1
E5–E9	16,300	18.7	Reference	—	—
O1–O3 [W1-W3]	4,458	18.7	1.0	1.0	1.0
O4–O10 [W4-W5]	2,864	19.5	1.1	1.0	1.1
Military occupation					
Combat-specific	6,044	19.7	1.1	1.1	1.1
Motor transport	1,452	20.2	1.1	1.1	1.2
Pilot / air crew	1,484	18.9	1.1	1.0	1.1
Repair / engineering	11,604	18.0	Reference	—	—
Communications / intelligence	8,841	18.6	1.0	1.0	1.1
Veterinarian	520	21.1	1.2	1.1	1.3
Health care	3,655	20.0	1.1	1.1	1.2
Other	8,952	21.2	1.2	1.1	1.2
Combatant command ^b^					
NORTHCOM	36,629				
CENTCOM	111				
INDOPACOM	2,407				
EUCOM	1,334				
AFRICOM	7				
SOUTHCOM	91				
Missing or unknown	1,973				

Abbreviations: No., number; IR, incidence rate; IRR, incidence rate ratio; CI, confidence interval; y, years; E, enlisted; O, officer; USPHS, U.S. Public Health Service; NORTHCOM, U.S. Northern Command; CENTCOM, U.S. Central Command; INDOPACOM, U.S. Indo-Pacific Command; EUCOM, U.S. European Command; AFRI-COM, U.S. Africa Command; SOUTHCOM, U.S. Southern Command.

a Service members were counted once per year.

b IR and IRR were not calculated for combatant commands, as person-time stratified by combatant command was not available.

Most bite and sting encounters reported from 2008 through 2023 occurred in the U.S., with 86.0% of bite and sting cases (n=36,629) in the U.S. Northern Command. The remainder of cases occurred in the U.S. Indo-Pacific Command (2,407 cases, 6%), U.S. European Command (n=1,334 cases, 3%), U.S. Southern Command (91 cases, >1), and U.S. Africa Command (7 cases, >1), while 5.0% (n=1,973) of cases were reported with missing or unknown locations (data not shown).

**FIGURE 1.**
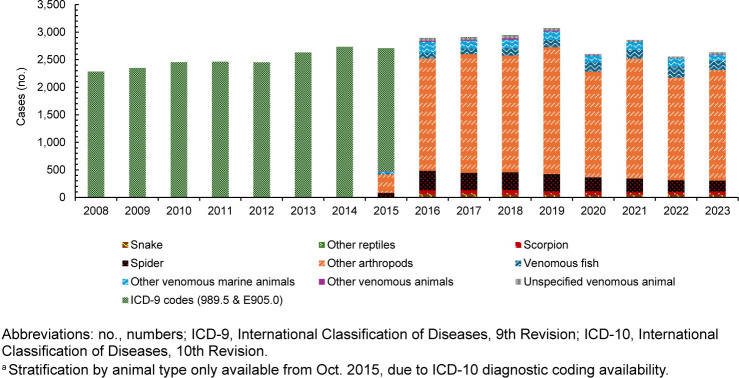
Annual Incident Cases of Venomous Bite and Sting Cases, by Type of Animal, Active Component U.S. Service Members, 2008–2023 ^a^

**FIGURE 2.**
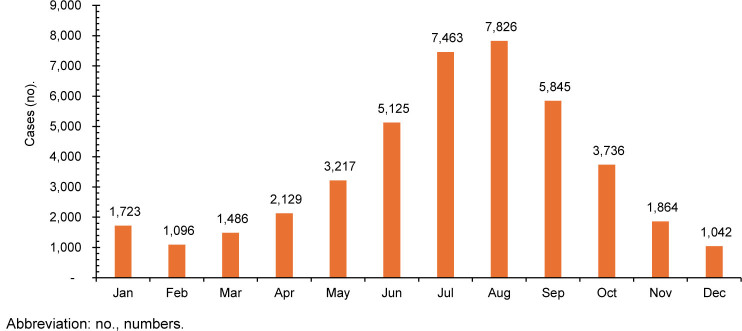
Total Number of Venomous Bite and Sting Cases by Month, Active Component U.S. Service Members, 2008–2023

The preponderance of bite and sting encounters reported (n=22,926) from October 2015 through 2023 was from other arthropods (75.0%), with 10.0% (n=2,281) from spider bites, 5.0% (n=1,178) from venomous fish, 5.0% (n=1,215) from other venomous marine animals, 2.0% (n=512) from scorpion stings, 2.0% (n=415) from snake bites, and 1.0% (n=303) from unspecified venomous animals, with 100 encounters from “other” venomous animals **(Figure 1)** . From 2008 through September 2015, there was no stratification by animal type in ICD-9 codes for bites and stings, and as a result, those encounters are reported cumulatively per year **(Figure 1)** . The distribution of incident cases throughout the surveillance period was also stratified by month to delineate case seasonality **(Figure 2)** . Most bites and stings occurred in the summer months (June–September), with August producing the greatest number of cases **(Figure 2)** .

Female service members showed an IR of 25 cases per 10,000 p-yrs compared to their male counterparts (18.2 cases per 10,000 p-yrs). Additionally, ACSMs younger than age 20 years had the highest incident rate (24.5 cases per 10,000 p-yrs). IRs were relatively high among non-Hispanic White and enlisted ACSMs **(Table 2)** . The IR was lowest within the Navy (13.5 cases per 10,000 p-yrs), albeit IRs were relatively similar among the other service branches **(Table 2)** .

**FIGURE 3.**
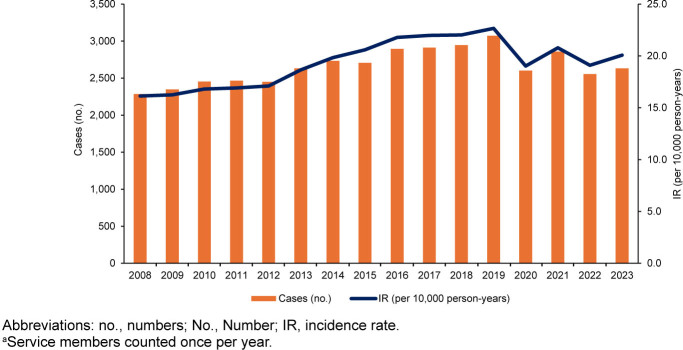
Annual Incidence Rates of Venomous Bite and Sting Cases ^a^ , Active Component U.S. Service Members, 2008–2023

Temporal analysis revealed a gradual increase in annual IR from 2008 (16.1 cases per 10,000 p-yrs) to a peak in 2019 (22.7 cases per 10,000 p-yrs), with a subsequent slight decrease in the final 4 years **(Figure 3)** of the surveillance period. The lowest and highest average rates occurred during the periods 2008–2012 (16.6 cases per 10,000 p-yrs) and 2016–2019 (22.1 cases per 10,000 p-yrs), respectively. A sharp decline followed the COVID-19 pandemic, dropping from 22.7 cases per 10,000 p-yrs in 2019 to 19.0 cases per 10,000 p-yrs in 2020 **(Figure 3)** .

The study also investigated IRRs to understand animal bite incident disparities within the ACSM population **(Table 2)** . The findings revealed significant differences in risk levels. Compared to men, women had approximately one-third higher risk of animal stings or bites during the period (IRR 1.37, 95% CI 1.3, 1,4). Age also emerged as a significant factor, with ACSMs younger than age 20 years exhibiting a 34% higher risk (IRR 1.3, 95% CI 1.3, 1.4) and those aged 45 years or older showing a 16% higher risk (IRR 1.16, 95% CI 1.1, 1.2) compared to the reference group of ACSMs aged 25-29 years.

Interestingly, occupation also played a role, as veterinarians and veterinary technicians were found 17.0% more likely to sustain an animal bite or sting (IRR 1.2, 95% CI 1.1, 1.3) than the reference group of repair and engineering military specialists **(Table 2)** .

## Discussion

Bites and stings from various animals pose a significant risk to ACSMs, with the data from 2008 through 2023 confirming this vulnerability. Although the majority of cases from 2008 through 2023 were among male service members of specific demographic and occupational groups, the IR was, unexpectedly, higher among women. This finding reflects, at least in part, the over-representation of women in veterinary roles (63.0% of veterinarians ^14^ and 88.0% of veterinary technicians ^15^ in the U.S. are women), exposing them to increased animal contact and occupational hazards.

The observed peak in IR during 2019 could be attributed to increased outdoor activities and operational tempo, followed by a decline coinciding with the COVID-19 pandemic's impacts on training and deployments. This analysis indicates that the majority of service member bites and stings from animals occur in the U.S., peaking during summer months **(Figure 2)** , when snakes, insects, and other animals are most active. ^8 - 10 , 16 , 17^ This is coincident with the higher risk faced by ACSMs engaged in warm weather field training exercises in wooded areas, particularly from arachnids such as the Black Widow spider ( *Latrodectus mactans* ). ^17^

Comparison with other published data on the U.S. general population is limited, but it is likely that IRs of venomous animal bites and stings are higher among military personnel due to their increased exposure to high-risk environments. ^9^ Arthropod envenomation accounts for a higher percentage of morbidity and mortality than snake envenomation among ACSMs ^8 , 10^ as well as the civilian population, ^3^ and medically significant scorpion sting encounters are fourth among ACSMs ^10^ and account for a high prevalence among the civilian population as well. ^16^ The estimated global toll from snake bites in the civilian population is substantial, with approximately 5.4 million people bitten by snakes each year, resulting in 1.8–2.7 million cases of envenoming and 81,410–137,880 deaths. ^2^ Similar to previous findings of venomous snake bites in the military, this analysis also shows venomous stings and bites of ACSMs to be highest in the U.S. compared to other combatant commands. ^9^

These report findings have implications for military planning and operations, particularly in regions with high risks of venomous animal encounters. The development of clinical practice guidelines such as the Joint Trauma System's clinical practice guideline training for bites, stings, and envenomation, ^6^ is crucial in providing comprehensive guidance for snakebite management and reducing risk of complications. Field guides ^10 , 18 , 19^ and clinical practice guidelines ^6 , 20^ are available for the U.S. Armed Forces, which offer personal protective measures against snake, marine animal, arthropod, and insect bites and stings to minimize envenomation and prevent diseases with militarily significant effects. These field guides and guidelines identify potentially dangerous animals that bite and sting, describe their biology and characteristics, recognize the symptoms caused by their venom or bites or stings, provide preventive measures, and learn appropriate treatment measures after military service members are bitten or stung.

To reduce risk of envenomation from animals of interest in this study, individuals can take preventive measures such as wearing protective clothing, using insect repellents, and avoiding high-risk areas. Proper training and education on how to identify and avoid venomous animals can also help reduce risk of envenomation. Awareness of one's surroundings and avoiding reaching or stepping into dark or hidden areas can help prevent snake bites and arthropod stings. When in marine environments, protective gear such as wetsuits or booties, in addition to avoidance of touching or handling marine animals, can help prevent marine envenomation. If envenomation occurs, prompt medical attention and appropriate treatment are essential to minimize risk of complications and ensure positive outcomes.

This study's findings underscore the importance of continued education, training, and access to medical care in minimizing risk of bites and stings and ensuring the health and operational readiness of military personnel. These findings also highlight the importance of considering individual characteristics when developing and implementing safety protocols for those working with animals.

One of the limitations of this retrospective report is that some cases of venomous animal bites and stings may not have been reported or documented, potentially leading to under-estimation of true incidence. While the DMSS provides a comprehensive database of medical encounters among military personnel, it may not capture all cases of venomous animal bites and stings, especially in locations outside the U.S., leading to under-reporting. This possible under-reporting could be the contributing factor to the high IR in the U.S combatant commands compared to other locations. Moreover, medical support is readily available within the U.S. compared to other locations, which could result in more U.S. medical encounters regardless of bite or sting allergic reaction severity; ACSMs in locations outside the U.S., including operations, may only seek medical care when they have severe allergic reactions to bites or stings.

Another limitation to the study is the use of diagnostic codes that are prone to coding errors, as well as changes in ICD code changes form ICD-9 to ICD-10 that could lead to differences in incidence. ICD-9 coding for venomous bites and stings does not delineate encounters by type of animal, whereas ICD-10 codes provide details and stratification by animal type. As a result, data from October 2015 onwards provide a clear delineation of impacts by different types of bites and stings, making it easier to determine leading contributors to medical encounters among ACSMs.

Future research should focus on identifying strategies to reduce the incidence of bites and stings, particularly among high-risk groups and those deployed to regions with high risks of venomous animal encounters. The report emphasizes the importance of proper training, education, and access to medical care in minimizing risk of bites and stings and ensuring the health and operational readiness of military personnel. The results of this report also suggest that the military should consider implementing targeted prevention and education programs for service members at high risk of venomous animal bites and stings.

## References

[1] Weinstein SA , White J . Toxins from venoms and poisons . In: Bozue J , Cote CK , Glass PJ , eds. Medical Aspects of Biological Warfare . Borden Institute, U.S. Army Medical Center of Excellence ; 2018 : 415 - 459 . Accessed Jul. 17, 2025 . https://medcoe.army.mil/borden-tb-medical-as-pects-bio-war

[2] World Health Organization . Snake Envenoming . 2023 . Accessed Jul. 17, 2025 . https://www.who.int/news-room/fact-sheets/detail/snakebite-envenoming

[3] Erickson TB , Cheema N . Arthropod envenomation in North America . Emerg Med Clin North Am . 2017 ; 35 ( 2 ): 355 - 375 . doi: 10.1016/j.emc.2017.01.001 28411932

[4] Cunha SA , Dinis-Oliveira RJ . Raising awareness on the clinical and forensic aspects of jellyfish stings: a worldwide increasing threat . Int J Environ Res Public Health . 2022 ; 19 ( 14 ): 8430 . doi: 10.3390/ijerph19148430 35886286 PMC9324653

[5] Hornbeak KB , Auerbach PS . Marine envenomation . Emerg Med Clin North Am . 2017 ; 35 ( 2 ): 321 - 337 . doi: 10.1016/j.emc.2016.12.004 28411930

[6] Joint Trauma System Trauma Care Educational Program . Clinical Practice Guideline Training: Global Snake Envenomation Management. Center for Excellence in Trauma, U.S. Dept. of Defense . Accessed Jul. 17, 2025 . https://jts.health.mil/index.cfm/cpgs/cpgs

[7] Yuan IH , Golden DB . Wings and stings: hymenoptera on vacation . Ann Allergy Asthma Immunol . 2023 ; 130 ( 4 ): 429 - 437 . doi: 10.1016/j.anai.2023.01.017 36702244

[8] Armed Forces Health Surveillance Branch . Hospitalizations of soldiers for insect, lizard, and snake envenomations . MSMR . 1997 ; 3 ( 8 ): 9 - 10 . Accessed Jul. 17, 2025 . https://www.health.mil/reference-center/reports/1997/01/01/medical-surveillance-monthly-report-volume-3-number-8

[9] Clark LL , Oh GT , Stahlman S . Brief report: medical encounters for snakebite envenomation, active and reserve components, US Armed Forces, 2016-2020 . MSMR . 2021 ; 28 ( 6 ): 13 - 5 . Accessed Jul. 17, 2025 . https://www.health.mil/reference-center/reports/2021/06/01/medical-surveillance-monthly-report-volume-28-number-06 34379380

[10] Shiau DT , Sanders JW , Putnam SD , et al . Self-reported incidence of snake, spider, and scorpion encounters among deployed U.S. military in Iraq and Afghanistan . Mil Med . 2007 ; 172 ( 10 ): 1099 - 1102 . doi: 10.7205/milmed.172.10.1099 17985774

[11] Turbyville JC , Dunford JC , Nelson MR . Hymenoptera of Afghanistan and the central command area of operations: assessing the threat to deployed US service members with insect venom hypersensitivity . Allergy Asthma Proc . 2013 ; 34 ( 2 ): 179 - 184 . doi: 10.2500/aap.2013.34.3638 23484895

[12] Dunford JC , Turbyville JC , Leavengood JM . Checklist of medically important hymenoptera of Afghanistan . Insecta Mundi . 2014 ; 0339 . Accessed Jul. 17, 2025 . https://journals.flvc.org/mundi/article/view/0339/79665

[13] Dunford JC , Kronmann KC , Peet LR , Stancil JD . Honey bee swarms aboard the USNS Comfort: recommendations for sting prevention, swarm removal, and medical readiness on military ships . US Army Med Dep J . 2016 ; 3-16 : 29 - 37 . Accessed Jul. 17, 2025 . https://medcoe.army.mil/the-medical-journal-archive 27613207

[14] U.S. Bureau of Labor Statistics . Veterinarians. Occupational Outlook Handbook. U.S. Dept. of Labor . Accessed Jul. 17, 2025 . https://www.bls.gov/ooh/healthcare/veterinarians.htm

[15] U.S. Bureau of Labor Statistics . Veterinary Technologists and Technicians. Occupational Out-look Handbook. U.S. Dept. of Labor . Accessed Jul. 17, 2025 . https://www.bls.gov/ooh/healthcare/veterinary-technologists-and-technicians.htm

[16] Kumar A , Goyal S , Garg MK , Gopalakrishnan M . Scorpion sting envenomation, a neglected tropical disease: a nationwide survey exploring perspectives and attitudes of resident doctors from India . Am J Trop Med Hyg . 2023 ; 109 ( 4 ): 957 - 964 . doi: 10.4269/ajtmh.23-0194 37696517 PMC10551078

[17] Kubena BE , Umar MA , Walker JD , Harper H . Case report: soldier with latrodectism after black widow spider bite during a field training exercise . Mil Med . 2023 ; 188 ( 3-4 ): e870 - e874 . doi: 10.1093/milmed/usab201 34027976

[18] Bowles LC , Swaby CJ . Field Guide to Venomous and Medically Important Invertebrates Affecting Military Operations: Identification, Biology, Symptoms, Treatment . Version 2.0. U.S. Air Force Institute for Operational Health ; 2006 .

[19] Armed Forces Pest Management Board . Technical Guide 36: Personal Protective Measures Against Insects and Other Arthropods of Military Significance. 2015. Office of the Under Secretary of Defense (Acquisitions and Sustainment), U.S. Dept. of Defense . Accessed Jul. 17, 2025 . https://www.acq.osd.mil/eie/afpmb/docs/techguides/tg36.pdf

[20] Joint Trauma System Trauma Care Educational Program . Clinical Practice Guideline Training: Bite, Sting, and Envenomation. Center for Excellence in Trauma, U.S. Dept. of Defense . Accessed Jul. 17, 2025 . https://jts.health.mil/assets/docs/cpgs/training/bites_stings_envenomation_cpg_training_09_feb_2021.pdf

